# Associations of time to the operating room on outcomes in odontogenic infection

**DOI:** 10.1186/s12903-024-05300-8

**Published:** 2025-01-20

**Authors:** Jeffrey N. James, Ryan Bloomquist, Kiara Brown, Stephen Looney, Dylan Walker, Tyler Day

**Affiliations:** 1 Louisiana State University Health New Orleans, LSUHSC School of Dentistry, 1100 Florida Avenue, New Orleans, LA 70119 USA; 2 University of South Carolina School of Medicine, Columbia, SC 29209 USA; 3 Dental College of Georgia, Augusta University, Augusta, GA 30904 Georgia

**Keywords:** Odontogenic Infection, Time to operating room, Oral and Maxillofacial Surgery

## Abstract

The purpose of this study was to determine if there were correlations between the length of time from hospital admission to surgical intervention and the frequency of complications in patients with odontogenic infections. While odontogenic infection is well studied in terms of interventions and outcomes, less is known about hospital utilization and resource burden of odontogenic infection with respect to timeliness to intervention. A retrospective cohort analysis was used to examine correlations between time from admission to surgical intervention and clinical outcomes. Patients included in this study were divided into three categories of length of time to the operating room: 0–12 h, 12.1–24 h, and greater than 24 h. Time of admission, time of surgical intervention, patient demographics, admission lab values, and space involvement were measured and compared to the primary outcome variables including complications of intubation attempts and type, ICU admission, length of hospitalization, number of changes in antibiotic therapy, and frequency of return to the operating room. We found that the length of time to the OR had a statistically significant association with length of hospital stay (*p* = 0.003) and number of changes in antibiotic therapy (*p* = 0.033). While overall length of hospital stay is inherently dependent on length of time to the OR, this relationship highlights the importance of timeliness to definitive intervention in order to reduce hospital burden. This study provides evidence on how to prioritize odontogenic infections in a hospital setting. We recommend treating odontogenic infection in less than 24 h from the time of admission in order to reduce costs and improve outcomes for patients.

## Introduction

Diagnosis and treatment of odontogenic infections are arguably among the most critical responsibilities of the oral and maxillofacial surgeon [1]. While cases of odontogenic infection are often non-emergent, they are pervasive in society and without timely intervention some have the potential to rapidly progress to life-threating illness [2]. Although the morbidity associated with odontogenic infections has gone down with the advent of antibiotics and improvements in healthcare, the number of odontogenic infections presenting in the emergency department has paradoxically gone up [3–5]. In the United States dental conditions made up approximately 1% of 400 million emergency department visits between 2008 and 2010, and abscess and cellulitis resulted in some of the highest costs and morbidities in this category [6]. Representation of the burden of odontogenic infections can vary depending on the healthcare system, and from 2000 to 2007, odontogenic infections made up 9.2% of emergency outpatient unit visits in Hamburg, Germany [7]. 

A frequent occurrence in both the developed and developing world, odontogenic infections can have severe localized and systemic effects. At the level of the oral cavity, they can precipitate severe pain, swelling and fever that can be debilitating. Left untreated, odontogenic infections can progress from localized infections to facial cellulitis and more concerningly into space infections and potentially death. Owing to the proximity of the viscerocranium to vital structures, dental infections may rapidly spread and develop into potentially life-threatening sequences such as Ludwig’s angina, cavernous sinus thrombosis, descending necrotizing mediastinitis, cerebral abscess, thoracic empyema, sepsis, or osteomyelitis [8, 9]. 

The effects of interventions on outcomes in odontogenic infections have been studied but it is a complex topic, as there are many confounders to consider. For example, one study found that there were significant impacts on education and whether or not a health care provider had been seen prior to presentation to the hospital on hospital course [10]. Furthermore, infections themselves are becoming more complex as our aging population becomes sicker and bacteria are becoming more resistant to antibiotic coverage [4, 11, 12]. Therefore, analysis of specific elements of odontogenic infection can give insight into a complex topic, such as the hospital burden associated with different approaches to intervention.

Aside from the potential mortality and morbidities that can result from untreated odontogenic infection, the prevalence of these cases can place a large burden on the healthcare system [13, 14]. While the number of uninsured has decreased following the Affordable Care Act, the overall financial burden of odontogenic infections remains high. Patients with odontogenic infections can cost hospital centers upwards of millions of dollars per year, and one study found the cost accrued by the hospital during this 8-year period was greater than $3.3 million at an average of $28,841 per patient, whereas the uncompensated charges submitted over this period were more than $10 million [15, 16]. Factors known to definitively increase hospital costs include operating room use, increasing patient medical complexity, length of stay, and increasing antibiotic resistance [14, 16]. One study found that the average length of stay for hospital admission resulting from odontogenic infection was 4.5 days, which resulted in substantial expense for the hospital system [15]. 

In this analysis, we set out to understand how the time from admission to surgical intervention impacted variables that could affect both patient outcomes and the frequency of complications in patients with odontogenic infections. We employed a retrospective chart review of patients admitted to the hospital for odontogenic infection. This information will help the oral and maxillofacial surgeon and emergency departments make evidence-based decisions on the management of odontogenic infection and is important for determining outcomes in earlier versus later surgical intervention.

## Materials and methods

### Study design

In this retrospective cohort study, we examined charts of 106 eligible patients with odontogenic infections treated at Augusta University by the Oral and Maxillofacial Surgery Department between July 1, 2015, through July 1, 2018 out of the 828 patients managed by the service in the hospital during that time period. Patients included in this study were divided into three categories of the predictor variable length of time to the operating room: 0–12 h, 12.1–24 h, and greater than 24 h. The patients’ charts were used to examine several independent outcome variables including time of admission, time to surgical intervention, admission lab values, to outcomes including intubation difficulty and type, ICU admission, length of hospital stay, changes in antibiotic therapy over the course of admission, and any returns to the OR. Additionally, parameters including smoking status, comorbidities, allergies to antibiotics, and treatment before arrival at Augusta University Hospital were considered. Primary inclusion included patients with a primary diagnosis of odontogenic infection that ultimately underwent surgical treatment at Augusta University Hospital during the study time period. Age was not considered as an exclusion criterion and those infections that did not require surgical intervention as well as those that developed infection as a secondary diagnosis to their admission were excluded. Patients were all treated and managed by the same Oral and Maxillofacial Department at Augusta University during the study time period.

### Data collection

Patients who were diagnosed and treated as inpatients at Augusta University Hospital from July 1, 2015, to July 1, 2018 for odontogenic infections were included in the study. Patients were excluded if their chart did not include information regarding all the studied variables. The study was approved the Institutional Review Board (IRB) at Augusta University, protocol number 1323780-3 and informed consent to participate was waived by this ethics committee. All experiments were performed in accordance with relevant guidelines and regulations.

## Statistical analysis

Categorical data were summarized using frequencies and percentages. Continuous data were summarized using mean ± standard deviation (S.D.), median, minimum, and maximum. Fisher’s exact test was used to compare groups in terms of categorical variables. Time to surgical treatment of infection was categorized as 0–12 h, 12.1–24 h, and > 24 h. These time points were chosen because of their practical implications. When managing odontogenic infection, the service on call may choose to take the case to the OR immediately, post it for later in the day, or post for the following day. Due to the high degree of skewness in the continuous variables, the median test was used to compare groups in terms of these variables, as single extreme outliers can significantly impact mean. For comparisons involving three groups, the median test was used to perform pairwise comparisons among groups using a Bonferroni-adjusted significance level of 0.05/3 = 0.0167. Spearman correlation was used to examine the association between continuous variables. All analyses were performed using SPSS software (Version 25, IBM Corp., 2017); all tests were two-tailed and were performed using a significance level of 0.05 unless otherwise indicated.

## Results

Data were available for a total of 106 eligible patients. Categorical patient characteristics and outcomes are summarized in Table 1. Continuous patient characteristics and outcomes are summarized in Table 2. Patients were placed into race categories of either black (50%), white (46%), or other/Hispanic (4%). Slightly more than half (54%) of the patients were female. The mean patient age was 38 years (median age = 34, range 4 to 89). 75% of the patients had trismus at admission. 16% were allergic to penicillin. The mean temperature at admission was 37^o^ C, and the mean white blood cell count (WBC) was 14. The mean number of comorbidities per patient was 1.1 (median 0, range 0 to 8). The mean number of spaces involved was 2.5 (median = 2.0, range 1 to 6). The mean time to surgical treatment was 15.6 h (median = 9.3, range 1 to 128.7). 17% of the patients were admitted to the ICU, and 6% had to return to the OR following the initial surgery. The most common type of intubation was video laryngoscopy (31%), followed by fiber-optic (27%) and direct laryngoscopy (26%). 11% of the patients required multiple intubation attempts. Almost half (46%) of the patients required only 1 antibiotic, but 35% required 2 or more. The type of antibiotic was changed in 15% of the patients. The mean hospitalization length of stay was 3.7 days (median = 2.5, range 0.5 to 61.3).

**Table 1:** Summary of categorical patient characteristics and outcomes

Characteristic	Number	Percent
Race		
Black	53	50.0
Other/Hispanic	4	3.8
White	49	46.2
Gender		
Female	57	53.8
Male	49	46.2
Trismus at Admission	79	74.5
Allergy to Penicillin	17	16.0
Return to OR	6	5.7
Admission to ICU	18	17.0
Type of Intubation (*n* = 104)		
Fiber Optic	28	26.9
Glidescope	32	30.8
Direct Laryngoscopy	27	26.0
Other	6	5.8
Multiple attempts	11	10.6
Antibiotic Category (*n* = 103)		
Change in Antibiotics Type	15	14.6
Single Antibiotic Therapy	47	45.6
Combined Antibiotics	36	35.0
Change in Antibiotics to Combined Therapy	5	4.9

**Table 2:** Summary of continuous patient characteristics and outcomes

Outcome	*n*	Mean	S.D.	Median	Minimum	Maximum
Age	106	38.3	15.9	34.0	4	89
Temperature at Admission	106	37.0	0.5	37.0	35.7	38.5
WBC count at Admission	105	14.0	4.8	13.8	5.6	28.9
Number of Spaces Involved	106	2.5	1.1	2.0	1	6
Comorbidities	106	1.1	1.5	0.0	0	8
Time to Surgical Treatment	106	15.6	19.8	9.3	1.0	128.7
Hospitalization Length of Stay	106	3.7	6.0	2.5	0.5	61.3

Prior to analysis, time to surgical treatment of infection was categorized as 0–12 h.

(*n* = 61), 12.1–24 h (*n* = 31), and > 24 h (*n* = 14). The three categories were then compared in terms of patient characteristics at baseline and in terms of outcomes. These results are summarized in Tables 3 and 4.

**Table 3:** Patient characteristics categorized by Time to Surgical Treatment

Characteristic	Time to Surgical Treatment			*p*-value
	0–12 h	12.1–24 h	> 24 h	
n	61	31	14	---
Race				
Black	25 (41%)	20 (65%)	8 (57%)	0.140
Hispanic	2 (3%)	1 (3%)	1 (7%)	
White	34 (56%)	10 (32%)	5 (36%)	
Gender				0.164
Female	28 (46%)	19 (61%)	10 (71%)	
Male	33 (54%)	12 (39%)	4 (29%)	
Age at Admission, mean ± SD	36.6 ± 12.7	40.0 ± 20.5	42.4 ± 17.5	0.924
Trismus at Admission	45 (74%)	24 (77%)	10 (71%)	0.903
Allergy to Penicillin	6 (10%)	7 (23%)	4 (29%)	0.081
Temperature at Admission, mean ± SD	37.0 ± 0.5	37.0 ± 0.5	37.0 ± 0.4	0.597
WBC count at Admission, mean ± SD (*n* = 104)	14.7 ± 4.5	13.3 ± 5.0	12.3 ± 4.9	0.530
Number of Spaces Involved, median (range)	3 (1–5)	2 (1–6)	2 (1–4)	0.005*
Comorbidities, median (range)	0 (0–8)	1 (0–4)	1.5 (0–5)	0.018*
Type of Intubation (*n* = 103)				0.297
Fiber Optic	18 (30%)	9 (30%)	1 (8%)	
Glidescope	18 (30%)	9 (30%)	5 (38%)	
Direct Laryngoscopy	16 (26%)	9 (30%)	2 (15%)	
Other	3 (5%)	2 (7%)	1 (8%)	
Multiple attempts	6 (10%)	1 (3%)	4 (31%)	

**Table 4:** Outcomes categorized by Time to Surgical Treatment

Outcome	Time to Surgical Treatment			*p*-value
	0–12 h	12.1–24 h	> 24 h	
Return to OR	5 (8%)	1 (3%)	0 (0%)	0.588
Admission to ICU	13 (21%)	4 (13%)	1 (7%)	0.468
Antibiotic Category (*n* = 102)				0.033*
Any Change in Antibiotics	10 (17%)	3 (10%)	7 (54%)	
Single Antibiotic Therapy	27 (45%)	17 (57%)	3 (23%)	
Combined Antibiotics	23 (38%)	10 (33%)	3 (23%)	
Hospitalization Length of Stay, mean ± SD	3.0 ± 2.2	3.1 ± 1.8	7.8 ± 15.5	0.003*

*Statistically significant (*p* < 0.05)

Patients in the three categories of time to surgical treatment did not differ significantly at in terms of age, race, gender, trismus, allergy to penicillin, temperature, WBC count, or type of intubation. The three categories did differ significantly at baseline in terms of the number of spaces involved (*p* = 0.005) and the number of comorbidities (*p* = 0.018). Using the Bonferroni-adjusted significance level of 0.0167, those patients in the 0–12 h category had significantly more spaces involved than in the 12.1–24 h category (*p* = 0.001). Those patients in the > 24 h category had significantly more comorbidities than the 0–12 h category (*p* = 0.006). There were no significant differences between the 12.1–24 h and > 24 h categories at baseline.

The three categories of time to surgical treatment of infection did not differ significantly in terms of return to the OR or admission to the ICU (Table 4). However they did differ significantly in terms of hospitalization length of stay (*p* = 0.003). Patients in the > 24 h category had a significantly greater length of stay than both the 0–12 h category (*p* = 0.001) and the 12.1–24 h category (*p* = 0.004) (Table 3). Because of the small frequency (*n* = 5) in the “Change in Antibiotics to Combined Therapy” antibiotics category (Table 1), this category was combined with the “Change in Antibiotics Type” (*n* = 15) to give a combined n of 20 for a new category “Any Change in Antibiotics.” This resulted in a significant association between the categorized time to surgical treatment of infection and antibiotic category (*p* = 0.033). A much larger percentage of patients in the > 24 h category (54%) experienced a change in antibiotics than in both the 0–12 h category (17%) and 12.1–24 h category (10%). A much smaller percentage had single antibiotic therapy in the > 24 h category (23% vs. 45% and 57%, respectively) (see Table 2).

The association between WBC count and length of hospitalization stay was weak but statistically significant (r_s_ = 0.217, *p* = 0.026). The association between comorbidities and length of stay almost reached statistical significance (r_s_ = 0.172, *p* = 0.078) (Table 3). Length of stay did not differ significantly between those who had pre-operative trismus and those who did not (*p* = 0.373) (Table 3). Trismus was not significantly associated with return to the OR (*p* = 1.000) or placement in the ICU (*p* = 0.553) (Tables 3 and 4). WBC count (*p* = 0.030) and number of spaces involved (*p* = 0.028) were significantly greater among those patients who returned to the OR (Table 3). The comparison of those patients who were placed in the ICU vs. those who were not in terms of WBC count did not quite reach statistical significance (*p* = 0.052) (Table 3). There were no other statistically significant associations or differences.

## Discussion

The purpose of this study was to determine how delayed treatment of odontogenic infections can affect outcomes variables. We hypothesized that delayed surgical intervention would lead to generally poorer outcomes. This study has the potential to impact the way that oral and maxillofacial surgeons and other healthcare professionals prioritize these infections in terms of proceeding to the operating room. Developing a standard time to the operating room may improve overall treatment outcomes as well as decrease the burden on the healthcare system caused by this disease process. We found that the length of time to the OR (surgical treatment) had a statistically significant association with only two of the primary outcomes; the overall length of hospital stay and the number of changes in antibiotic therapy, with the strongest differences between the group with time to surgical intervention > 24 h versus < 12 h.

In addition to these primary outcomes, we found a statistically significant association between time to surgical treatment and the number of spaces involved. This could be attributed to progression of the infection immediately prior to surgical intervention when the infection is uncontrolled and potentially rapidly progressing. The number of patient comorbidities was found to be significantly correlated with time to surgical treatment as well. This may be attributed to compounding underlying comorbidities that the patient had at the time of admission allowing a more rapid spread of infection due to immunocompromising conditions. Due to these variables, it is difficult to determine if the increased length of hospital stay and changes in antibiotic regimen are more attributable to the delayed surgical intervention or to other factors which, in and of themselves, may coincidentally delay taking patients to the OR. We did not find a strong statistically significant association between preexisting health conditions and length of hospital stay. However, other studies have shown that increased preexisting health conditions do in fact increase hospital stay [17]. Benefits could be gained from future studies that may be able to control for such factors and determine the true relationship between time to operating room and the primary outcomes in the study.

It is important to highlight confounders and limitations in the interpretation of this data. Inherent to this analysis is the association between severity if infection, number of spaces involved, and ultimately the time to OR. Here, severe and rapidly progressing infections are triaged and prioritized in how fast they are taken to the OR, thus creating a confounder in the association of the predictor and outcomes. Complex odontogenic infections can progress rapidly and result in mortality making a controlled trial involving delay to OR for complex infections unfeasible. Therefore, this study is retrospective in nature and associations but not causation can be determined. However, it is also important to note that while more advanced infections necessitate earlier intervention, this early and definitive treatment results in an overall shorter stay in the hospital even though the infections are more severe. Another important point to discuss is a timing bias relative to timing of intervention. As this analysis reports on the predictor variable of time to the OR at 0–12-hour, 12–24 h, or greater than 24 h, a timing bias of intervention must be discussed. Here, a longer time prior to intervention (i.e. greater than 24 h), will inherently lead to longer time at the hospital. While acknowledging this bias, this reinforces the importance of a definitive intervention for odontogenic infections through surgery, as delay to treatment results in an overall increase in hospital stay, including the time period prior to the intervention, resulting in increased cost, utilization, and morbidity burden to the patient and hospital system.

Not considered in this study is the time that the patient took to seek treatment. Oftentimes, patients, particularly uninsured patients, have attempted to seek care elsewhere, and have been given antibiotics prior to their worsening symptoms and their ultimate arrival at a hospital. Additional research is needed in this area to determine the effects of total time to treatment and the overall burden on the hospital.

Additionally, although the study was able to demonstrate that a delay in treatment of odontogenic infections led to increased length of hospital stays, the study was unable to provide actual cost data in terms of concrete dollar amounts per patient. Important to be considered in future studies would be to analyze the costs, benefits, and practicality of implementing a standard time to the operating room for these odontogenic infection patients.

Our analysis gives evidence regarding the outcomes of early versus late intervention from hospital admission for dental infections. Here, we find that treating the patient in under 24 h is associated with both a reduction in the length of stay for the patient as well as a reduction in the number of changes in antibiotics type, an indicator of a reduction in infection progression. We recommend that odontogenic infection be treated within a 24-hour period for better patient outcomes and in order to reduce costs and burdens to the hospital system.

## Data Availability

All data and materials are available within this manuscript.
